# Comparison of various doses of oral cannabidiol for treating refractory epilepsy indications: a network meta-analysis

**DOI:** 10.3389/fneur.2024.1243597

**Published:** 2024-06-27

**Authors:** Xin Wang, Haiyan Zhu, Tao Liu, Zhi Guo, Chenyang Zhao, Zhiyi He, Wenxu Zheng

**Affiliations:** ^1^The First Affiliated Hospital of China Medical University, Shenyang, China; ^2^Department of Neurosurgery, Tianjin Medical University General Hospital, Tianjin, China; ^3^Geriatric Department of Dalian Friendship Hospital, Dalian, China

**Keywords:** seizure, refractory epilepsy, antiseizure drugs, cannabidiol, network meta-analysis

## Abstract

**Aim:**

To evaluate the comparative efficacy and safety of various doses of oral cannabidiol (CBD) in treating refractory epilepsy indications, thus providing more informative evidence for clinical decision-making.

**Methods:**

A literature search of PubMed, Embase, the Cochrane library, and Web of Science (WoS) was performed to retrieve relevant randomized controlled trials (RCTs) that compared different doses of oral CBD with placebo or each other in refractory epilepsy indications. The search was limited from the inception of each database to January 3, 2023. Relative risk [RR] with a 95% confidence interval [CI] was used to express results. STATA/SE 14 was employed for network meta-analysis.

**Results:**

Six RCTs involving 972 patients were included in the final data analysis. Network meta-analysis showed that, CBD10 (10 mg/kg/day) (RR: 1.77, 95%CI: 1.28 to 2.44), CBD20 (20 mg/kg/day) (RR: 1.91, 95%CI: 1.49 to 2.46), CBD25 (25 mg/kg/day) (RR: 1.61, 95%CI: 0.96 to 2.70), and CBD50 (50 mg/kg/day) (RR: 1.78, 95%CI: 1.07 to 2.94) were associated with higher antiseizure efficacy although the pooled result for CBD25 was only close to significant. In addition, in terms of the risk of treatment-emergent adverse events (TEAEs), the difference between different doses is not significant. However, CBD20 ranked first in terms of antiseizure efficacy, followed by CBD50, CBD10, and CBD25. For TEAEs, CBD25 ranked first, followed by CBD10, CBD50, CBD5, and CBD20.

**Conclusion:**

For refractory indications, CBD20 may be optimal option for antiseizure efficacy; however, CBD25 may be best for TEAEs. Therefore, an appropriate dose of oral CBD should be selected based on the actual situation. Due to the limitations of eligible studies and the limited sample size, more studies are needed in the future to validate our findings.

## Introduction

Epilepsy is one of the most frequently happening neurological disorders (1), which has complex pathological causes, such as structural, metabolic, genetic, infectious, and immune factors (2). In addition, the etiologies causing seizures remain unclear in approximately 50% of cases (3). Among patients with epilepsy, there is about 30% occurrence rate of drug-refractory seizures, which cannot be treated with the current anti-epileptic drugs (AEDs) (4). Refractory epilepsy is developed from epilepsy and refers to the use of two or more antiepileptic drugs (monotherapy or combination therapy) for treatment, but the seizure cannot be controlled (5). Therefore, it is also known as drug-resistant epilepsy or refractory epilepsy. According to the International League Against Epilepsy, drug-refractory epilepsy refers to “failure of ≥2 appropriate and tolerated AEDs to achieve the sustained freedom of seizures” (6). Failure in seizure control will bring a higher risk of brain damage, injury, and death (7, 8), and the risk is extraordinarily high for patients with refractory epileptic syndromes such as Dravet syndrome (DS), tuberous sclerosis complex (TSC), and Lennox–Gastaut syndrome (LGS) (9). DS and LGS are severe, treatment-resistant developmental epileptic encephalopathies (DEEs), in which seizure activity is associated with general cerebral dysfunction (9). TSC is a genetic neurocutaneous disorder with epilepsy as a common and early presenting symptom (10).

Current treatments for epileptic seizures generally aim to increase inhibition or decrease hyperexcitation in the neurotransmission pathways related to seizure pathogenesis (11, 12). However, many AEDs, such as Clobazam, do not show adequate effect in many patients (13). These AEDs could not work as an intervention to control refractory seizures by targeting different intercellular pathways (14). Scientists found that Cannabis (CNB) is effective to enhance seizure control for patients with epilepsy that cannot be treated with existing AEDs. They have investigated substitutive treatments with CNB derivative compounds for refractory epilepsy (15, 16). Two major ingredients in CNB is Δ9-tetrahydrocanabidiol (THC) for psychotropic functions (17), and cannabidiol (CBD) for medical effects in epilepsy (18, 19). Both CNB extracts with THC and CBD are effective in treating refractory epilepsy indications (20). Compared to THC, CBD lacks psychoactive properties and does not lead to dependence or abuse (21).

Cannabinol has anti-epileptic properties, and its anti-epileptic mechanism is mainly manifested in the following aspects. First, it may be related to genetic changes in ion channels. Cannabidiol may selectively inhibit sodium current reflux, reduce neuronal excitability, and exert anticonvulsant effects by altering the expression of epilepsy related Nav1.6 mutated sodium channels (22, 23). Second, CBD can act as allosteric modulators on CBR1, altering the ortho signal of G protein coupled receptors, thereby inhibiting synaptic plasticity (24). Third, CBD is an antagonist of cannabinoid receptor GPR55, and when combined, it can increase GABA signaling and reduce excitatory neuronal discharge (25). At last, CBD may also be related to the agonist and antagonist effects of some receptors (22). In 2018, the FDA officially approved CBD as an additional antiepileptic drug, mainly for patients aged 2 years and above with Ds and LGS (26).

Although CBD’s effects on the brain have not been fully understood (21, 27), the therapeutic effect of CBD in refractory epilepsy indications has been evaluated in some clinical studies (28–30). In addition, many meta-analyses (14, 31–35) have also established the therapeutic potential of oral CBD in the treatment of refractory epilepsy indications (e.g., LGS, DS, and CST). At present, various recommended doses of oral CBD were available in clinical practice. A recent meta-analysis showed 10, 20, and 50 mg/kg/day were all effective for treating refractory epilepsy indications (14), indicating that the effect and tolerance of oral CBD may be not dose-dependent. However, other studies found that 5 and 25 mg/kg/day had significant antiseizure efficacy, and thought 25-mg/kg/day dosage had a better safety profile than the 50-mg/kg/day dosage (36, 37). Hence, this network meta-analysis aimed to determine the optimal dose of oral CBD for the treatment of refractory epilepsy indications. In addition, we must need to know that several factors such as the type of epilepsy, age, and other concomitant molecules were not considered adequately by previous meta-analysis. As a result, findings of the previous meta-analyses did not actually inform practitioners which dose of oral CBD may be preferred for treating refractory epilepsy (14, 31–35). Therefore, this network meta-analysis fully considered the impact of some influencing factors (e.g., type of epilepsy, age, and other concomitant molecules) on the therapeutic efficacy and safety.

## Materials and methods

### Study design

The current network meta-analysis strictly followed the Preferred Reporting Items for Systematic Reviews and Meta-Analyses (PRISMA) for network meta-analysis (PRISMA-NMA) (38). Due to the nature of the data extracted from published studies in this network meta-analysis, it was unnecessary to involve ethics approval or informed consent. However, we must point out that the protocol of this network meta-analysis was not registered on any public platforms.

### Inclusion and exclusion criteria

Studies that met the following criteria were included: (a) studies included patients who were diagnosed with refractory epilepsy; (b) comparison had been made among different doses of oral CBD or between different doses of oral CBD and placebo; (c) therapeutic efficacy and safety were reported; (d) randomized controlled trials (RCTs) were published in English.

We excluded studies: (a) used ineligible study designs, such as case reports, conference abstracts, retrospective studies, or observational studies; (b) focused on other neurological disorders; (c) recruited fewer than 10 participants; (d) had insufficient data to allow for evaluation of efficacy and safety; (e) were comprised of partially overlapping patient populations.

### Data search

Two independent authors (Xin Wang and Haiyan Zhu) performed a systematic literature search in PubMed, Embase, the Cochrane library, and Web of Science (WoS) databases to retrieve potentially eligible randomized controlled trials (RCTs) from their inception to January 3rd, 2023. We constructed a search strategy by combining the following search terms and their analogs: “drug resistant epilepsy,” “seizures,” “epilepsy,” “Lennox Gastaut Syndrome,” “Myoclonic Epilepsies,” “Tuberous Sclerosis,” “cannabidiol,” and “cannabis.” The search strategies for all target databases are documented in Supplementary Table S1. In addition, we have also manually checked the reference lists of previous meta-analyses and eligible studies to find additional studies missed from the electronic search.

### Study selection

Study selection was performed by two independent authors (Tao Liu and Zhi Guo) based on the inclusion and exclusion criteria. All records from electronic databases were first imported into EndNote software (version X9) to develop a literature database, and the software automatically eliminated duplicate records. Then, two authors (Chenyang Zhao and Zhiyi He) screened the titles and abstracts of each study for the initial eligibility assessment, and they excluded the ineligible studies. We further assessed the full texts of the remaining studies to determine which studies may be eligible for selection criteria. The consensus principle based on the discussion was employed to resolve disagreements between these two authors (Haiyan Zhu and Wenxu Zheng).

### Data extraction

Two independent authors (Xin Wang and Tao Liu) extracted the following information from each eligible study using a standardized data extraction form: the name of the first author, publication time, country, sample size, the percentage of male patients, average age, indications, the number of the patients who taken concomitant AEDs and clobazam, the details of orally taking CBD, study duration, and outcomes of interest. In addition, detailed information on the risk of bias in each study was also extracted at this stage. For those results that were reported as median and standardized error or interquartile range (IQR), we transformed it to the required data by using the recognized formulas (39).

### Definitions of outcomes

Based on the previous traditional pair-wise meta-analysis (14), this network meta-analysis designed two outcomes to evaluate the efficacy and safety of oral CBD for refractory epilepsy indications. Specifically, the therapeutic efficacy was evaluated by using at least 50% reduction in seizure frequency relative to the baseline seizures reported. For this outcome, the intention-to-treat (ITT) data were used for statistical analysis. In addition, the safety of oral CBD was defined based on the treatment-emergent adverse events (TEAEs) observed and reported in the trials.

### Evidence plot

We constructed some evidence plots to show the evidence structure of all outcomes evaluated in this network meta-analysis. Two essential elements were involved in each evidence plot, including a solid circle and line. A solid circle represents an oral dose of CBD, and a solid line represents a direct comparison between the two doses of CBD. In addition, the accumulated number of patients for each dose was employed to weigh the size of the circle, and the accumulated number of direct comparisons between the two doses was used to weigh the width of the solid line.

### Risk of bias assessment

Two independent authors (Zhi Guo and Chenyang Zhao) used the revised Risk of Bias Assessment Tool (ROB2) (40) to assess the risk of bias in each eligible study. Specifically, the risk of bias assessment was performed according to the following six bias domains: randomization process; deviations from intended intervention; missing outcome data; measurement of the outcome; and selection of the reported result. The overall risk of bias in each study was determined based on the result of each domain, and each study was labeled with a “low,” “high,” or “some concerns” risk. Ultimately, the overall results of the risk of bias assessment were graphically presented using an online application, namely ‘robvis’ (41).

### Statistical analysis

All outcomes in this network meta-analysis were dichotomous variables, therefore, we used the relative risk (RR) with the corresponding 95% confidence interval (CI) to express the effect size. Statistical heterogeneity was evaluated using the Cochrane *Q* statistic and Higgins’ inconsistency factor (*I^2^*), and significant statistical heterogeneity exist if *p* < 0.1 and *I^2^* > 50% (42). However, we did not directly compare different doses with placebo because a recent pair-wise meta-analysis including the same studies has evaluated the relative efficacy and safety of various doses of oral CBD compared to placebo, indicating an insignificant statistical heterogeneity for available comparisons (14).

We assessed the transitivity assumption by comparing the distribution of five clinical and methodological variables (43), including the percentage of males, mean age, the number of concomitant AEDs, the percentage of patients who orally take clobazam, and treatment duration. After confirming the transitivity assumption, we performed random effects network meta-analysis (White).

We first tested the global inconsistency by comparing the result from the command “network meta i” with the result from the command “network meta c” (44). In addition, we also tested the local inconsistency by using the node-splitting strategy (45). We selected an appropriate network meta-analysis model according to inconsistency examination results.

Furthermore, we calculated the SUCRA value to rank all doses of oral CBD, and the larger SUCRA value means a higher probability of becoming the preferred option (46). When there was a closed loop for the outcome, we also used the node-splitting strategy to assess the loop inconsistency which means whether direct effect was equal to indirect effect in a closed loop, thereby assessing the reliability of pooled results (47, 48). Although the number of eligible studies did not exceed 10, we still drew comparison-adjusted funnel plots to test the risk of the small-study effect (49). Finally, we generated a cluster plot to help determining which oral dose may be the optimal option comprehensively. We used STATA/SE 14.0 (StataCorp, Texas, USA) to perform all statistical analyses in this network meta-analysis.

## Results

### Literature selection

**Figure 1:**
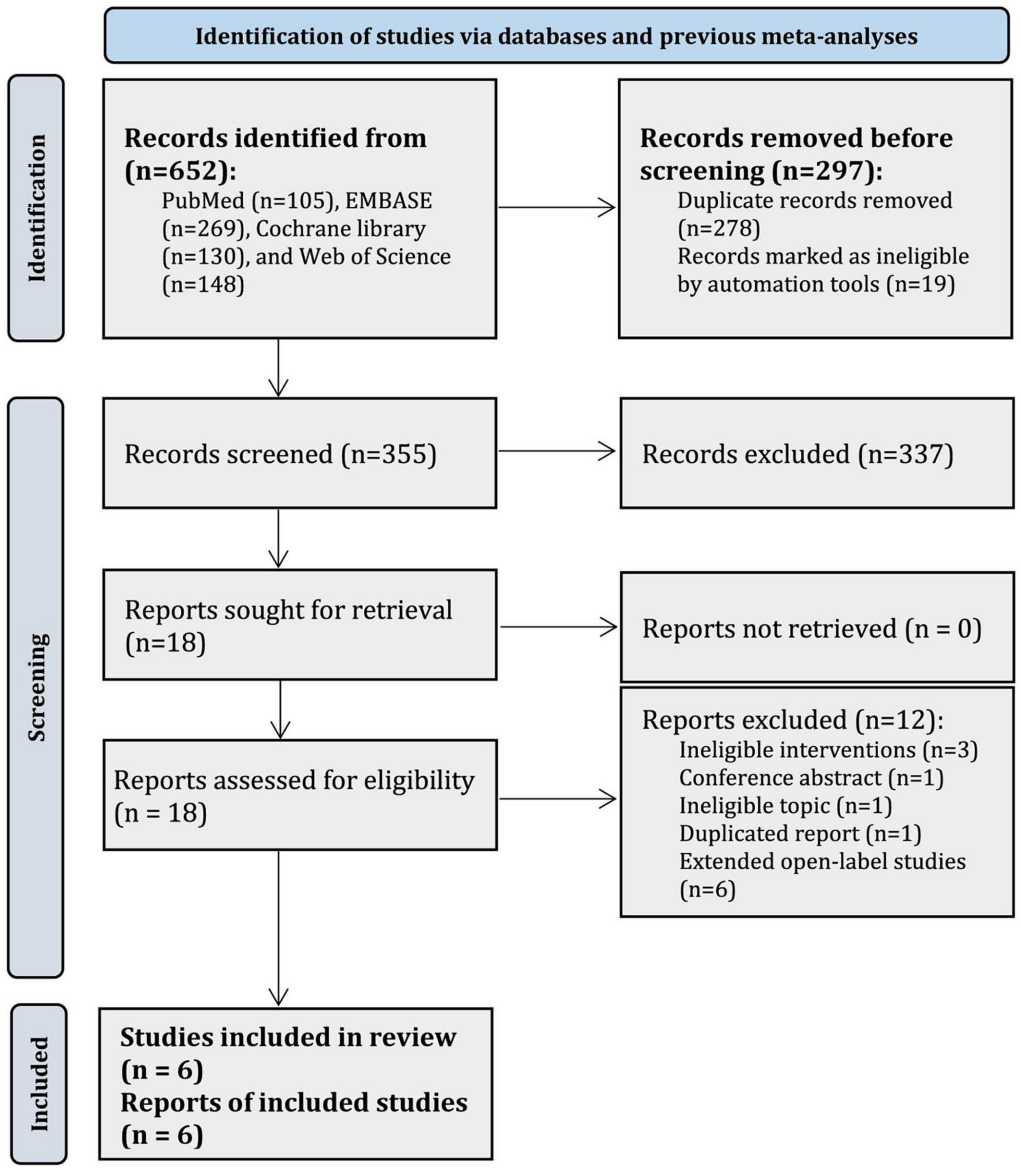
PRISMA flow chart for the selection of eligible studies.

In total, 652 records were identified in PubMed (*n* = 105), EMBASE (*n* = 269), Cochrane library (*n* = 130), and WoS (*n* = 148), and 278 duplicate records and 19 registry records were removed by using EndNote software. After abstract screening, 337 records were further excluded. Thus, the remaining 18 articles were initially judged as potentially relevant. Among them, we further excluded 12 studies due to ineligible interventions (*n* = 3), conference abstract (*n* = 1), ineligible topic (*n* = 1), duplicated report (*n* = 1), and extended open-label studies (*n* = 6). Finally, 6 RCTs (28–30, 36, 37, 50) were included in the current network meta-analysis. The flow chart for eligible studies screening is presented in Figure 1.

### Study characteristics

The basic characteristics of 6 eligible studies are summarized in Table 1. All eligible studies were published between 2017 and 2021. Three studies (28, 36, 50) assessed pediatric and adolescent patients, but the other three studies (29, 30, 37) also involved adult patients under the age of 57 years. In total, 972 patients with refractory epileptic syndromes were accumulated finally, with 368 patients in the placebo group, 10 patients in the CBD5 group, 151 patients in the CBD10 group, 307 patients in the CBD20 group, 75 patients in the CBD25 group, and 73 patients in the CBD50 group. The average age of patients analyzed in all eligible studies was 11.1 years. Of these six studies, five (28–30, 37, 50) reported therapeutic efficacy; however, all six studies (28–30, 36, 37, 50) reported the data on therapeutic safety. Regarding CBD5, CBD25 and CBD50, only one study assessed the therapeutic efficacy, respectively, and most patients were male. Regarding CBD25, three studies were involved, and the proportion of male were 38.0, 55.0, and 41.0%, respectively. In terms of CBD20, five studies were involved, and most participants were male. As shown in Supplementary Table S2, the transitivity assumption was basically confirmed in most available comparisons except for the comparisons of CBD20 with CBD25 and CBD50.

**Table 1:** Basic characteristics of studies included in this network meta-analysis (*n* = 6).

Study	Country	Group	Details of interventions	Sample size	Males, %	Age, years		Indication	No. of concomitant AEDs	No. of Clobazam	Study duration
						Mean	Rang				
(28)	USA	Placebo	Patients received oral CBD at 20 mg/kg/day or matched placebo.	59	46.0	9.8	2.3–18.4	DS	2.9 ± 1.0	38	18 weeks
		CBD20		61	57.0	9.7			3.0 ± 1.0	40	
(29)	USA	Placebo	Patients received oral CBD at dose 5, 10, or 20 mg/kg/day or matched placebo.	7	71.0	7.0	4–10	DS	2.1 ± 0.9	5	14 weeks
		CBD5		10	50.0	7.2			2.6 ± 1.1	6	
		CBD10		8	38.0	7.4			2.8 ± 0.5	6	
		CBD20		9	33.0	8.7			2.8 ± 0.8	6	
(36)	USA	Placebo	Patients receive oral CBD at 10 or 20 mg/kg/day, or matched placebo.	76	58.0	15.3	2–55	LGS	3.0 ± 1.0	37	14 weeks
		CBD10		73	55.0	15.4			3.0 ± 1.0	37	
		CBD20		76	59.0	16.0			3.0 ± 1.3	36	
(30)	USA	Placebo	Patients received oral CBD at 20 mg/kg/day or matched placebo.	85	51.0	15.3	2–55	LGS	3.0 ± 0.8	43	14 weeks
		CBD20		86	52.0	15.5			3.0 ± 1.0	41	
(50)	USA	Placebo	Patients received CBD oral solution at 10 or 20 mg/kg/day or matched placebo.	65	48.0	9.6	2–18	DS	3.0 ± 1.0	41	14 weeks
		CBD10		66	41.0	9.2			3.0 ± 1.0	45	
		CBD20		67	54.0	9.3			3.0 ± 0.8	40	
(37)	USA	Placebo	Patients received oral CBD at 25 or 50 mg/kg/day or a matched placebo.	76	59.0	10.9	1.1–56.8	TSC	3.0 ± 1.0	25	16 weeks
		CBD25		75	57.0	11.6			3.0 ± 0.8	17	
		CBD50		73	59.0	10.2			3.0 ± 1.0	19	

### Risk of bias assessment

We assessed the risk of bias of the six eligible RCTs, and the detailed results are depicted in Supplementary Figure S1. All studies were judged as having low risk in the randomization process, deviations from intended interventions, missing outcome data, and selection of the reported result. But all studies had some concerns in the measurement of the outcome due to the lack of detailed information on whether the outcome assessor were blinded or not. The overall risk of bias in each eligible study was rated as “low,” because outcomes that we were interested in had not been significantly affected by the outcome assessors.

### Network meta-analysis

#### Therapeutic efficacy

**Figure 2:**
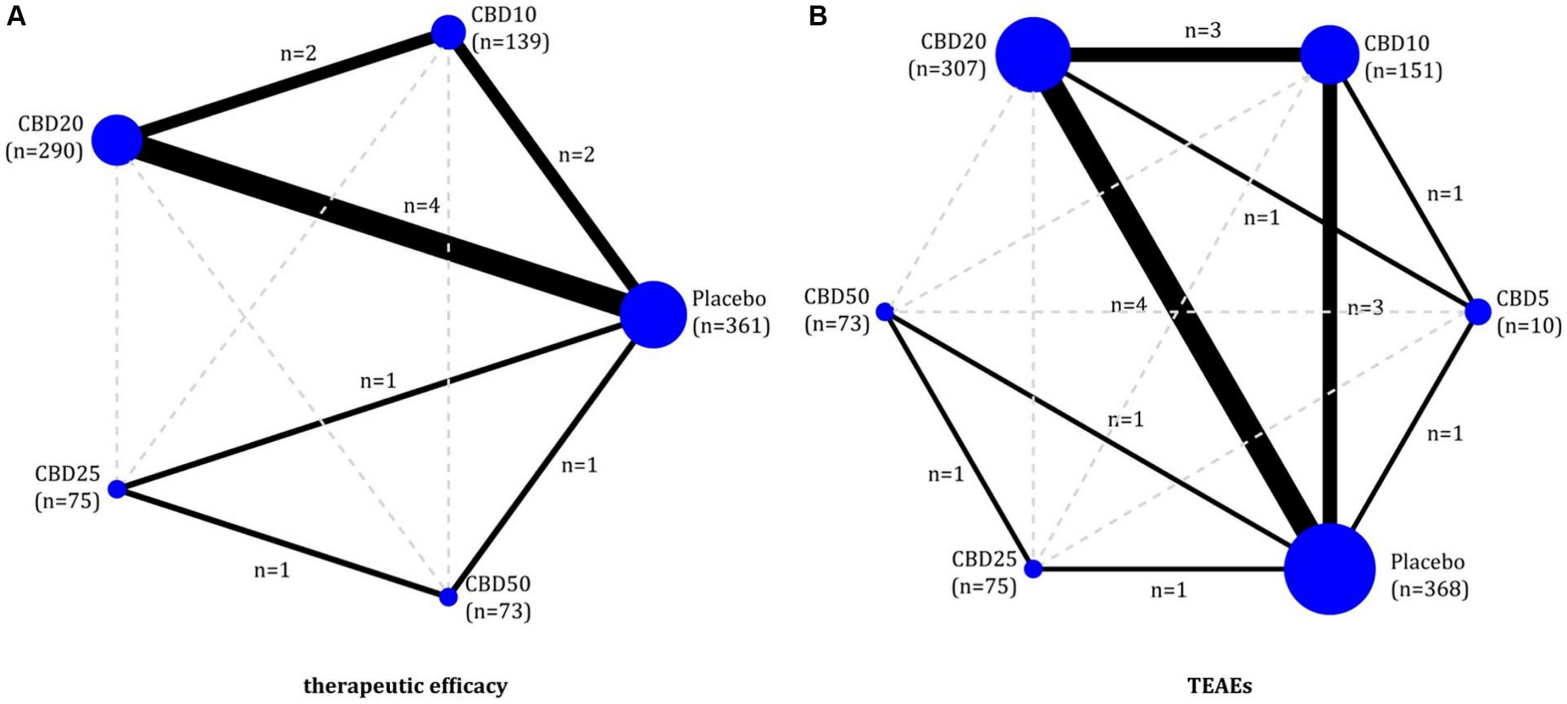
Evidence plots of therapeutic efficacy **(A)** and TEAEs **(B)**. CBD, cannabidiol; TEAEs, treatment-emergent adverse events.

**Figure 3:**
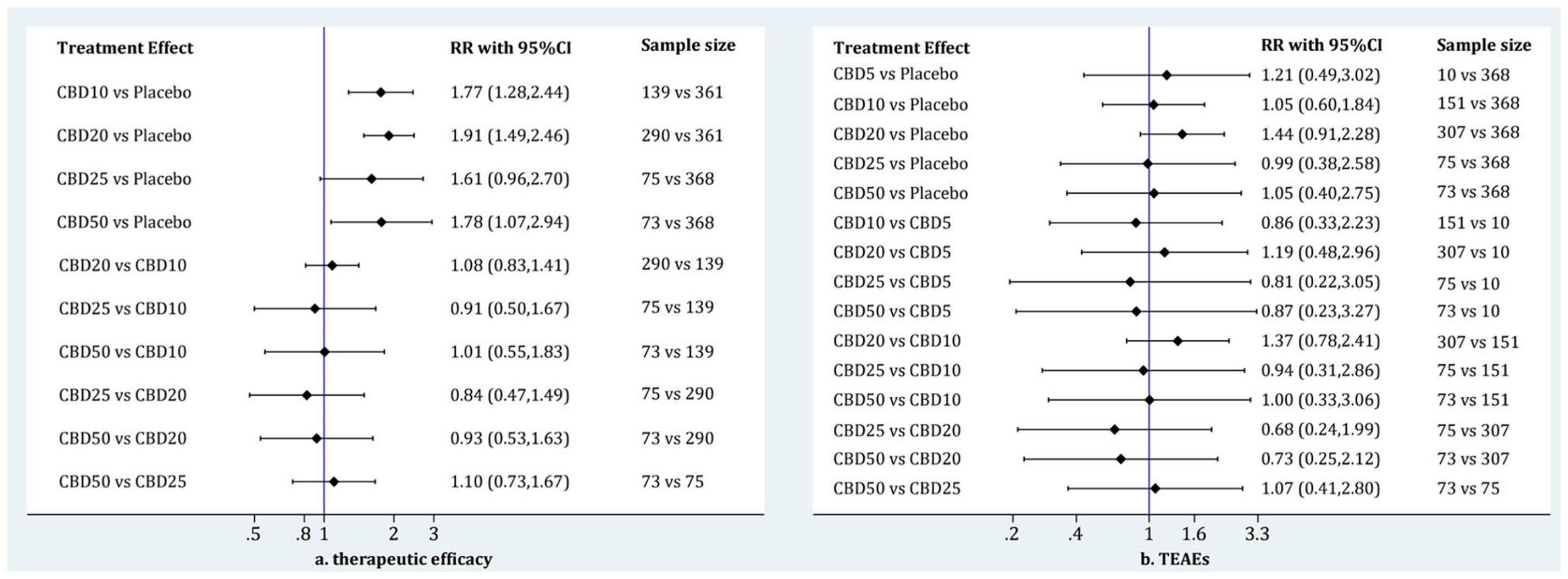
The results of network meta-analysis of therapeutic efficacy **(A)** and TEAEs **(B)**. CBD, cannabidiol; TEAEs, treatment-emergent adverse events; RR, relative risk; CI, confidence interval.

Of 6 eligible studies, five (11, 17, 18, 22, 32) reported the data on therapeutic efficacy involving four doses of oral CBD, including CBD10, CBD20, CBD25, and CBD50. As shown in Figures 2A, 6 two-by-two direct comparisons were available for this outcome. As shown in Supplementary Figure S2a, global inconsistency for the therapeutic efficacy was not detected (*p* = 0.399), and local inconsistency was also not found (as shown in Supplementary Figure S3a). Therefore, the consistency model was employed for the network meta-analysis of therapeutic efficacy. As shown in Figure 3A, compared to placebo, CBD10 (RR: 1.77, 95%CI: 1.28–2.44), CBD20 (RR: 1.91, 95%CI: 1.49–2.46), CBD25 (RR: 1.61, 95%CI: 0.96–2.70), and CBD50 (RR: 1.78, 95%CI: 1.07–2.94) were associated with higher antiseizure efficacy although the pooled result for CBD25 was only close to significant. However, there was no significant difference between these four oral doses of CBD based on the currently available data.

#### Short-term therapeutic safety

All six eligible RCTs (11, 17, 18, 22, 32, 51)reported the TEAEs to reflect the short-term therapeutic safety, involving five doses of oral CBD, including CBD5, CBD10, CBD20, CBD25, and CBD50. As shown in Figure 2B, 8 two-by-two direct comparisons were available for this outcome. Inconsistency tests did not detect global (*p* = 0.486) and local inconsistency (as shown in Supplementary Figure S3b). So, we employed the consistency model for the network meta-analysis of TEAEs. As shown in Figure 3B, all available doses of oral CBD did not significantly increase the risk of TEAEs as compared with placebo, and there was no significant difference in the risk of TEAEs between all available doses.

### Probability ranking

**Figure 4:**
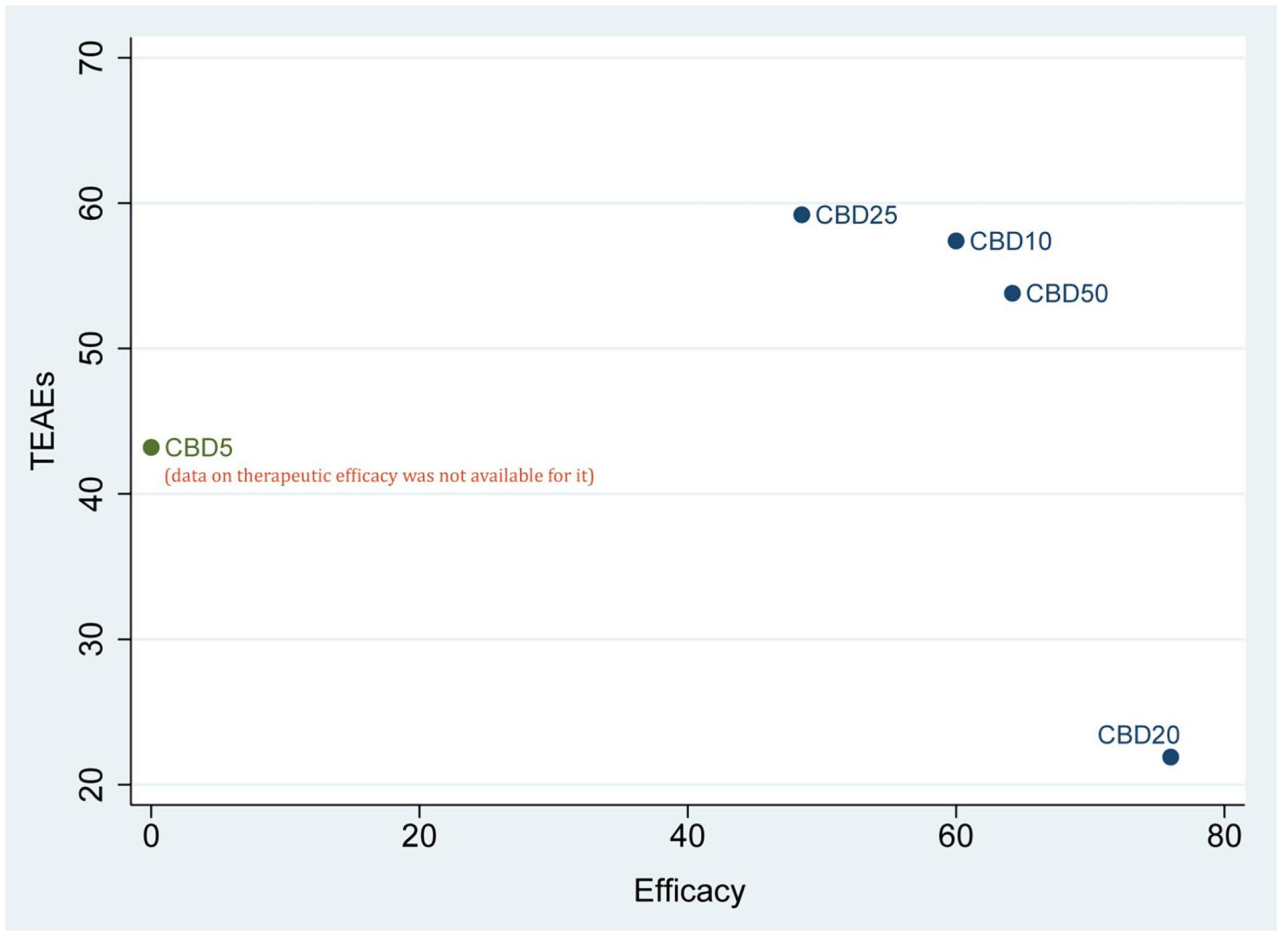
Cluster plot of combining the SUCRA values for therapeutic efficacy and TEAEs. CBD, cannabidiol; TEAEs, treatment-emergent adverse events.

According to the results of SUCRA values, CBD20 had the highest probability of becoming the optimal option (76.0%) for the therapeutic efficacy, followed by CBD50 (64.2%), CBD10 (60.0%), and CBD25 (48.5%). However, regarding short-term safety (TEAEs), CBD25 ranked first, with the highest probability of 59.2%, followed by CBD10 (57.4%), CBD50 (53.8%), CBD5 (43.2%), and CBD20 (21.9%). As shown in Figure 4, the cluster plot combining the ranking probability for therapeutic efficacy and TEAEs showed the overall distribution of the optimal dose.

### Loop inconsistency

**Figure 5:**
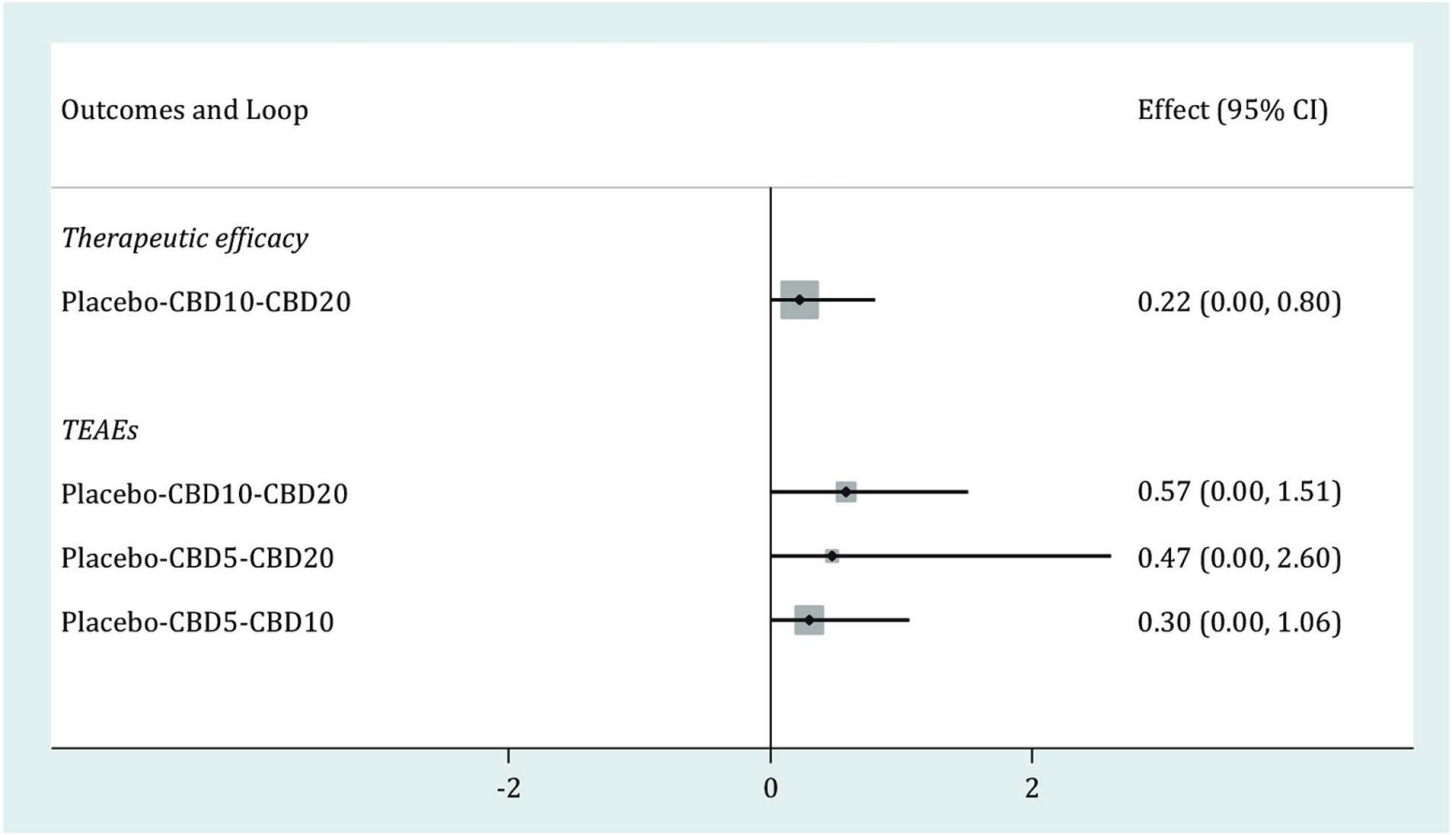
Loop inconsistency test for therapeutic efficacy and TEAEs. CBD, cannabidiol; TEAEs, treatment-emergent adverse events; CI, confidence interval.

As shown in Figure 5, one closed loop (Placebo-CBD10-CBD20) was available in therapeutic efficacy, and three closed loops (Placebo-CBD10-CBD20, Placebo-CBD5-CBD20, Placebo-CBD5-CBD10,) were available in TEAEs. The results of the loop inconsistency test for these two outcomes showed that the lower limits of all 95% CIs were zero, thus suggesting no inconsistency for all available loops in these two outcomes.

### Publication bias

The comparison-adjusted funnel plots of the therapeutic efficacy (a) and TEAEs (b) are shown in Supplementary Figure S4. According to the visual inspection of these two funnel plots, we speculate that publication bias might negatively impact the reliability and robustness of these two outcomes.

## Discussion

To our best knowledge, this is the first study to determine the optimal dose of oral CBD for treating refractory epilepsy indications by employing the network meta-analytic technique. Finally, we included 6 eligible RCTs in the final data analysis, involving a total of 972 patients. According to the pooled results, we find that, compared to placebo, CBD5, CBD10, CBD20, CBD25 were associated with higher antiseizure efficacy although the pooled result for CBD25 was only close to significant. This was because only one study (37) assessed the antiseizure efficacy of CBD 25. Furthermore, our results reported that various doses of oral CBD did not differ in the risk of TEAEs. The results of SUCRA showed that CBD20 may be the optimal option for the therapeutic efficacy but worst option for safety. However, regarding TEAEs, CBD25 may be the best option.

The current network meta-analysis considered the impact of sex differences on the antiseizure efficacy because previous studies (51–53) have shown that epileptic seizures and antiseizure response to some AEDs are sex-specific. Specifically, men are usually more susceptible to excitability episodes and occurrence of epileptic seizures than women, but women are more often diagnosed with idiopathic systematic epilepsy than men. However, the most recently meta-analysis (14) reported that the currently available evidence only provided little insight about the age-specific differences of CBD in anti-seizure response. In the current network meta-analysis, transitivity assessment showed that the distribution of gender ratio (males/females) did not significantly differ between various dosage strategies of CBD, indicating that our findings will not be negatively influenced by sex differences. Nevertheless, our network meta-analysis could not also determine the sex-specific anti-epileptic effect and safety of CBD because the gender ratio is evenly distributed. Therefore, more studies should be performed to investigate sex-specific differences of CBD in anti-epileptic response and safety.

The prevalence of active epilepsy varies with age and shows a bimodal distribution, peaking in children aged 5–9 and the elderly aged over 80 (2019). Therefore, apart from sex differences, age is also considered to have an impact on anti-epileptic response and safety. It is worth noting that the current network meta-analysis showed that the mean age of patients evenly distributed between different dosage strategies, indicating that our results will not be negatively affected by this factor. However, it should not be ignored that some eligible studies evaluated patients in a wider age range. Therefore, it is necessary to recruit comparable patients to further investigate age-specific effect of CBD on refractory epilepsy indications.

To date, several meta-analyses (14, 31–35) have evaluated oral CBD’s therapeutic potential in treating patients with refractory epilepsy indications, and all showed that oral CBD was associated with significantly higher antiseizure efficacy. Furthermore, the meta-analysis performed by Talwar et al.(14) evaluated the therapeutic efficacy of various doses of oral CBD on refractory epilepsy indications by using subgroup analysis, showed that, except for 25 mg/kg/day, 10, 20, and 50 mg/kg/day were all associated with significantly higher antiseizure efficacy. Compared with previous meta-analyses, the current study employed network meta-analysis to compare doses of oral CBD, therefore determined the possible best dose of oral CBD for the treatment of refractory epilepsy indications. In addition, the current network meta-analysis combined direct and indirect evidence simultaneously to generate more accurate estimation of the efficacy of each dose on refractory epilepsy. However, the pooled result for CBD25 in our network meta-analysis was only close to significant, which was consistent with the finding of the previous meta-analysis performed by Talwar and colleagues (14). According to a cohort study (54), CBD25 was effective for treating children with refractory epilepsy. We need to point out that, in this network meta-analysis, only 75 patients were accumulated in the CBD25 group, therefore insufficient sample size may explain why the pooled result was just close to significant.

Our network meta-analysis also consistently showed that 10, 20, 25, and 50 mg/kg/day benefited refractory epilepsy although the benefit of CBD25 to refractory epilepsy was close to statistically significant; however, the difference between these available doses of oral CBD did not achieve a significant level. The recent meta-analysis (14) suggested that, due to very limited understanding of pharmacodynamics and mechanism of action, therefore it is extremely difficult to explain why different doses of oral CBD did not differ in efficacy. However, due to the fact that patients with refractory epilepsy typically receive multiple drugs for treatment, it is speculated that the adjunct effects with other AEDs and complex drug interactions can lead to different antiseizure efficacies (8), which is why the interaction of CBD and clobazam is especially emphasized (55–57). In fact, in this network meta-analysis, we detected that the percentage of patients who orally took clobazam in the CBD20 group was significantly more than that in the CBD25 and CBD50 groups. Therefore, we speculate that the concomitant use of more clobazam may contribute to the higher antiseizure efficacy but lower safety in the CBD20 group. In addition, we also found no significant difference in the percentage of patients who orally took clobazam between CBD10 and CBD20. However, the SUCRA values showed that the ranking probability of CBD20 was higher than CBD10 in therapeutic efficacy but lower in safety, thereby providing promising evidence to support that higher dose is associated with greater seizure control but also with higher AEs (58).

We must emphasize that this network meta-analysis generates some valuable findings for decision-making due to the involvement of several methodological strengths. First, our network meta-analysis included only RCTs in the final analysis to enhance the power of the evidence. Second, both direct and indirect data were incorporated to estimate the relative difference between various oral doses of CBD more accurately. Third, we employed the results of SUCRA to perform a probability ranking of all available doses of oral CBD, which provides more informative evidence for determining the preferred dose.

We also need to acknowledge several limitations in this network meta-analysis. First and foremost, only six studies were included in the final analysis. So, it is extremely difficult to generate robust and reliable results due to the limited eligible studies and sample size, thereby noting us to cautiously interpret findings. Second, although the available dose of oral CBD ranged from 5 to 50 mg/kg/day, the therapeutic efficacy of 5 mg/kg/day was not evaluated in the included RCTs due to the data on this outcome was not available in the eligible studies, which inevitably compromised our findings’ applicability. Third, although the percentage of patients who orally took clobazam did not evenly distribute between some of the available doses, which may influence the therapeutic effects and safety, subgroup analysis has not been performed to further evaluate the impact on the pooled results due to limited eligible studies. Fourth, visual inspection for comparison-adjusted funnel plots detected publication bias, which may negatively influence the reliability of our findings (59). However, we need to point out that the number of studies included in this network meta-analysis does not meet the lowest criteria of performing a publication bias examination, so it is impossible to eliminate the negative impact of inadequate eligible studies on the test. Fifth, we only evaluated the short-term therapeutic safety. Thus, future studies should be performed to evaluate the long-term safety of various doses. Sixth, among the 6 included RCTs, three studies were performed by Devinsky et al. (28, 29, 36), and two studies were performed by Thiele et al. (30, 37), which may introduce bias to impact the reliability of the pooled results. Therefore, more studies are needed to further verify our findings. Finally, we did not publicly register the formal protocol for the current network meta-analysis, although we strictly followed the PRISMA-NMA statement the absence of formal protocol will inevitably compromise the transparency of our network meta-analysis.

Based on the currently available evidence, our findings indicated that CBD5, CBD10, CBD20, CBD25 were associated with higher antiseizure efficacy although the pooled result for CBD25 was only close to significant. Various doses of oral CBD did not differ in the risk of TEAEs. Furthermore, for refractory indications, CBD20 may be optimal option for antiseizure efficacy; however, CBD25 may be best for TEAEs. Therefore, an appropriate dose of oral CBD should be selected based on the actual situation. Due to the limitations of study quantity and sample size, future RCTs with larger sample sizes and high quality is warranted for the further validation of our findings.

## Data availability statement

The original contributions presented in the study are included in the article/Supplementary material, further inquiries can be directed to the corresponding author/s.

## Author contributions

XW and HZ carried out the studies, participated in collecting data, and drafted the manuscript. XW, TL, and ZH performed the statistical analysis and participated in its design. ZG, CZ, and WZ participated in acquisition, analysis, or interpretation of data and draft the manuscript. All authors read and approved the final manuscript.

## Supplementary material

SUPPLEMENTARY FIGURE S1 Detailed results of the risk of bias assessment for all eligible studies.

SUPPLEMENTARY FIGURE S2 Global inconsistency test for therapeutic efficacy **(a)** and TEAEs **(b)**. CBD, cannabidiol; TEAEs, treatment-emergent adverse events.

SUPPLEMENTARY FIGURE S3 Local inconsistency test for therapeutic efficacy and TEAEs. CBD, cannabidiol; TEAEs, treatment-emergent adverse events; CI, confidence interval.

SUPPLEMENTARY FIGURE S4 Comparison-adjusted funnel plot for therapeutic efficacy **(a)** and TEAEs **(b)**. TEAEs, treatment-emergent adverse events.

## References

[1] Wężyk K Słowik A Bosak M. Predictors of remission in patients with epilepsy. Neurol Neurochir Pol. (2020) 54:434–9. doi: 10.5603/PJNNS.a2020.0059 32757204

[2] Falco-Walter J . Epilepsy-definition, classification, pathophysiology, and epidemiology. Semin Neurol. (2020) 40:617–23. doi: 10.1055/s-0040-1718719, PMID: 33155183

[3] Tanaka A Akamatsu N Shouzaki T Toyota T Yamano M Nakagawa M . Clinical characteristics and treatment responses in new-onset epilepsy in the elderly. Seizure. (2013) 22:772–5. doi: 10.1016/j.seizure.2013.06.005, PMID: 23849689

[4] Kwan P Brodie MJ. Early identification of refractory epilepsy. N Engl J Med. (2000) 342:314–9. doi: 10.1056/nejm200002033420503 10660394

[5] Song L Liu F Liu Y Zhang R Ji H Jia Y. Clonazepam add-on therapy for refractory epilepsy in adults and children. Cochrane Database Syst Rev. (2018) 5:Cd012253. doi: 10.1002/14651858.CD012253.pub2, PMID: 29717488 PMC6494417

[6] Kwan P Arzimanoglou A Berg AT Brodie MJ Allen Hauser W Mathern G . Definition of drug resistant epilepsy: consensus proposal by the ad hoc task force of the ILAE commission on therapeutic strategies. Epilepsia. (2010) 51:1069–77. doi: 10.1111/j.1528-1167.2009.02397.x, PMID: 19889013

[7] Elmer S Reddy DS. Therapeutic basis of generic substitution of Antiseizure medications. J Pharmacol Exp Ther. (2022) 381:188–96. doi: 10.1124/jpet.121.000994, PMID: 35241634 PMC9132097

[8] Reddy DS . Therapeutic and clinical foundations of cannabidiol therapy for difficult-to-treat seizures in children and adults with refractory epilepsies. Exp Neurol. (2023) 359:114237. doi: 10.1016/j.expneurol.2022.114237, PMID: 36206806

[9] Chin RF Mingorance A Ruban-Fell B Newell I Evans J Vyas K . Treatment guidelines for rare, early-onset, treatment-resistant epileptic conditions: a literature review on Dravet syndrome, Lennox-Gastaut syndrome and CDKL5 deficiency disorder. Front Neurol. (2021) 12:734612. doi: 10.3389/fneur.2021.734612, PMID: 34759881 PMC8573384

[10] van der Poest Clement E Jansen FE Braun KPJ Peters JM. Update on drug Management of Refractory Epilepsy in tuberous sclerosis complex. Paediatr Drugs. (2020) 22:73–84. doi: 10.1007/s40272-019-00376-0, PMID: 31912454

[11] Kanner AM Bicchi MM. Antiseizure medications for adults with epilepsy: a review. JAMA. (2022) 327:1269–81. doi: 10.1001/jama.2022.3880 35380580

[12] Reddy DS . Clinical pharmacology and therapeutics of antiepileptic drugs for treatment of epilepsy and seizure disorders. Int. J. Pharmaceutical Sci. Nanotechnol. (2020) 13:5165–80. doi: 10.37285/ijpsn.2020.13.6.1

[13] Leahy JT Chu-Shore CJ Fisher JL. Clobazam as an adjunctive therapy in treating seizures associated with Lennox-Gastaut syndrome. Neuropsychiatr Dis Treat. (2011) 7:673–81. doi: 10.2147/ndt.S20173, PMID: 22128252 PMC3225341

[14] Talwar A Estes E Aparasu R Reddy DS. Clinical efficacy and safety of cannabidiol for pediatric refractory epilepsy indications: a systematic review and meta-analysis. Exp Neurol. (2023) 359:114238. doi: 10.1016/j.expneurol.2022.114238, PMID: 36206805

[15] Friedman D Devinsky O. Cannabinoids in the treatment of epilepsy. N Engl J Med. (2015) 373:1048–58. doi: 10.1056/NEJMra1407304 26352816

[16] O'Connell BK Gloss D Devinsky O. Cannabinoids in treatment-resistant epilepsy: a review. Epilepsy Behav. (2017) 70:341–8. doi: 10.1016/j.yebeh.2016.11.012 28188044

[17] Renard J Norris C Rushlow W Laviolette SR. Neuronal and molecular effects of cannabidiol on the mesolimbic dopamine system: implications for novel schizophrenia treatments. Neurosci Biobehav Rev. (2017) 75:157–65. doi: 10.1016/j.neubiorev.2017.02.006, PMID: 28185872

[18] Kaplan EH Offermann EA Sievers JW Comi AM. Cannabidiol treatment for refractory seizures in Sturge-weber syndrome. Pediatr Neurol. (2017) 71:e12:–23.e2. doi: 10.1016/j.pediatrneurol.2017.02.009 28454984

[19] Pazos MR Mohammed N Lafuente H Santos M Martínez-Pinilla E Moreno E . Mechanisms of cannabidiol neuroprotection in hypoxic–ischemic newborn pigs: role of 5HT1A and CB2 receptors. Neuropharmacology. (2013) 71:282–91. doi: 10.1016/j.neuropharm.2013.03.027, PMID: 23587650

[20] Pamplona FA Da Silva LR Coan AC. Potential clinical benefits of CBD-rich cannabis extracts over purified CBD in treatment-resistant epilepsy: observational data meta-analysis. Front Neurol. (2018) 9:759–767. doi: 10.3389/fneur.2018.00759 30258398 PMC6143706

[21] Devinsky O Cilio MR Cross H Fernandez-Ruiz J French J Hill C . Cannabidiol: pharmacology and potential therapeutic role in epilepsy and other neuropsychiatric disorders. Epilepsia. (2014) 55:791–802. doi: 10.1111/epi.12631, PMID: 24854329 PMC4707667

[22] Ibeas Bih C Chen T Nunn AV Bazelot M Dallas M Whalley BJ. Molecular targets of Cannabidiol in neurological disorders. Neurotherapeutics. (2015) 12:699–730. doi: 10.1007/s13311-015-0377-3, PMID: 26264914 PMC4604182

[23] Patel RR Barbosa C Brustovetsky T Brustovetsky N Cummins TR. Aberrant epilepsy-associated mutant Nav1.6 sodium channel activity can be targeted with cannabidiol. Brain. (2016) 139:2164–81. doi: 10.1093/brain/aww129, PMID: 27267376 PMC4958898

[24] Straiker A Dvorakova M Zimmowitch A Mackie K. Cannabidiol inhibits endocannabinoid signaling in Autaptic hippocampal neurons. Mol Pharmacol. (2018) 94:743–8. doi: 10.1124/mol.118.111864, PMID: 29669714 PMC5988021

[25] Kaplan JS Stella N Catterall WA Westenbroek RE. Cannabidiol attenuates seizures and social deficits in a mouse model of Dravet syndrome. Proc Natl Acad Sci USA. (2017) 114:11229–34. doi: 10.1073/pnas.1711351114, PMID: 28973916 PMC5651774

[26] Sanmartin PE Detyniecki K. Cannabidiol for epilepsy: new Hope on the horizon? Clin Ther. (2018) 40:1438–41. doi: 10.1016/j.clinthera.2018.07.020, PMID: 30150078

[27] Reddy DS . The utility of Cannabidiol in the treatment of refractory epilepsy. Clin Pharmacol Ther. (2017) 101:182–4. doi: 10.1002/cpt.441 27506704

[28] Devinsky O Cross JH Laux L Marsh E Miller I Nabbout R . Trial of Cannabidiol for drug-resistant seizures in the Dravet syndrome. N Engl J Med. (2017) 376:2011–20. doi: 10.1056/NEJMoa1611618 28538134

[29] Devinsky O Patel AD Cross JH Villanueva V Wirrell EC Privitera M . Effect of cannabidiol on drop seizures in the Lennox–Gastaut syndrome. N Engl J Med. (2018) 378:1888–97. doi: 10.1056/NEJMoa1714631 29768152

[30] Thiele EA Marsh ED French JA Mazurkiewicz-Beldzinska M Benbadis SR Joshi C . Cannabidiol in patients with seizures associated with Lennox-Gastaut syndrome (GWPCARE4): a randomised, double-blind, placebo-controlled phase 3 trial. Lancet. (2018) 391:1085–96. doi: 10.1016/S0140-6736(18)30136-3, PMID: 29395273

[31] Lattanzi S Brigo F Cagnetti C Trinka E Silvestrini M. Efficacy and safety of adjunctive Cannabidiol in patients with Lennox-Gastaut syndrome: a systematic review and Meta-analysis. CNS Drugs. (2018) 32:905–16. doi: 10.1007/s40263-018-0558-9, PMID: 30132269

[32] de Carvalho Reis R Almeida KJ da Silva Lopes L de Melo Mendes CM Bor-Seng-Shu E. Efficacy and adverse event profile of cannabidiol and medicinal cannabis for treatment-resistant epilepsy: systematic review and meta-analysis. Epilepsy Behav. (2020) 102:106635. doi: 10.1016/j.yebeh.2019.106635, PMID: 31731110

[33] Silvinato A Floriano I Bernardo WM. Use of cannabidiol in the treatment of epilepsy: Lennox-Gastaut syndrome, Dravet syndrome, and tuberous sclerosis complex. Rev Assoc Med Bras. (2022) 68:1345–57. doi: 10.1590/1806-9282.2022D689, PMID: 36417631 PMC9683917

[34] Treves N Mor N Allegaert K Bassalov H Berkovitch M Stolar OE . Efficacy and safety of medical cannabinoids in children: a systematic review and meta-analysis. Sci Rep. (2021) 11:23462. doi: 10.1038/s41598-021-02770-6, PMID: 34873203 PMC8648720

[35] Zhang L Wang J Wang C. Efficacy and safety of antiseizure medication for Lennox-Gastaut syndrome: a systematic review and network meta-analysis. Dev Med Child Neurol. (2022) 64:305–13. doi: 10.1111/dmcn.15072, PMID: 34590711

[36] Devinsky O Patel AD Thiele EA Wong MH Appleton R Harden CL . Randomized, dose-ranging safety trial of cannabidiol in Dravet syndrome. Neurology. (2018) 90:e1204–11. doi: 10.1212/wnl.0000000000005254, PMID: 29540584 PMC5890607

[37] Thiele EA Bebin EM Bhathal H Jansen FE Kotulska K Lawson JA . Add-on Cannabidiol treatment for drug-resistant seizures in tuberous sclerosis complex: a placebo-controlled randomized clinical trial. JAMA Neurol. (2021) 78:285–92. doi: 10.1001/jamaneurol.2020.4607, PMID: 33346789 PMC7754080

[38] Hutton B Salanti G Caldwell DM Chaimani A Schmid CH Cameron C . The PRISMA extension statement for reporting of systematic reviews incorporating network meta-analyses of health care interventions: checklist and explanations. Ann Intern Med. (2015) 162:777–84. doi: 10.7326/m14-2385, PMID: 26030634

[39] Wan X Wang W Liu J Tong T. Estimating the sample mean and standard deviation from the sample size, median, range and/or interquartile range. BMC Med Res Methodol. (2014) 14:135. doi: 10.1186/1471-2288-14-135, PMID: 25524443 PMC4383202

[40] Sterne JA Savović J Page MJ Elbers RG Blencowe NS Boutron I . RoB 2: a revised tool for assessing risk of bias in randomised trials. BMJ. (2019) 366:l4898. doi: 10.1136/bmj.l4898 31462531

[41] McGuinness LA Higgins JPT. Risk-of-bias VISualization (robvis): an R package and shiny web app for visualizing risk-of-bias assessments. Res Synth Methods. (2021) 12:55–61. doi: 10.1002/jrsm.1411, PMID: 32336025

[42] Higgins JP Thompson SG. Quantifying heterogeneity in a meta-analysis. Stat Med. (2002) 21:1539–58. doi: 10.1002/sim.1186, PMID: 12111919

[43] Cipriani A Higgins JP Geddes JR Salanti G. Conceptual and technical challenges in network meta-analysis. Ann Intern Med. (2013) 159:130–7. doi: 10.7326/0003-4819-159-2-201307160-00008, PMID: 23856683

[44] Higgins JP Jackson D Barrett JK Lu G Ades AE White IR. Consistency and inconsistency in network meta-analysis: concepts and models for multi-arm studies. Res Synth Methods. (2012) 3:98–110. doi: 10.1002/jrsm.1044, PMID: 26062084 PMC4433772

[45] Dias S Welton NJ Caldwell DM Ades AE. Checking consistency in mixed treatment comparison meta-analysis. Stat Med. (2010) 29:932–44. doi: 10.1002/sim.3767, PMID: 20213715

[46] Mbuagbaw L Rochwerg B Jaeschke R Heels-Andsell D Alhazzani W Thabane L . Approaches to interpreting and choosing the best treatments in network meta-analyses. Syst Rev. (2017) 6:79. doi: 10.1186/s13643-017-0473-z, PMID: 28403893 PMC5389085

[47] Lu G Ades AE. Assessing evidence inconsistency in mixed treatment comparisons. J Am Stat Assoc. (2006) 101:447–59. doi: 10.1198/016214505000001302

[48] Yu-Kang T . Node-splitting generalized linear mixed models for evaluation of inconsistency in network Meta-analysis. Value Health. (2016) 19:957–63. doi: 10.1016/j.jval.2016.07.005, PMID: 27987646

[49] Chaimani A Higgins JP Mavridis D Spyridonos P Salanti G. Graphical tools for network meta-analysis in STATA. PLoS One. (2013) 8:e76654. doi: 10.1371/journal.pone.0076654, PMID: 24098547 PMC3789683

[50] Miller I Scheffer IE Gunning B Sanchez-Carpintero R Gil-Nagel A Perry MS . Dose-ranging effect of adjunctive Oral Cannabidiol vs placebo on convulsive seizure frequency in Dravet syndrome: a randomized clinical trial. JAMA Neurol. (2020) 77:613–21. doi: 10.1001/jamaneurol.2020.0073, PMID: 32119035 PMC7052786

[51] Reddy DS Thompson W Calderara G. Molecular mechanisms of sex differences in epilepsy and seizure susceptibility in chemical, genetic and acquired epileptogenesis. Neurosci Lett. (2021) 750:135753. doi: 10.1016/j.neulet.2021.135753, PMID: 33610673 PMC7994197

[52] Christian CA Reddy DS Maguire J Forcelli PA. Sex differences in the epilepsies and associated comorbidities: implications for use and development of pharmacotherapies. Pharmacol Rev. (2020) 72:767–800. doi: 10.1124/pr.119.017392, PMID: 32817274 PMC7495340

[53] Reddy DS . The neuroendocrine basis of sex differences in epilepsy. Pharmacol Biochem Behav. (2017) 152:97–104. doi: 10.1016/j.pbb.2016.07.002, PMID: 27424276

[54] Sands TT Rahdari S Oldham MS Caminha Nunes E Tilton N Cilio MR. Long-term safety, tolerability, and efficacy of Cannabidiol in children with refractory epilepsy: results from an expanded access program in the US. CNS Drugs. (2019) 33:47–60. doi: 10.1007/s40263-018-0589-2, PMID: 30460546

[55] de Leon J Spina E Diaz FJ. Clobazam therapeutic drug monitoring: a comprehensive review of the literature with proposals to improve future studies. Ther Drug Monit. (2013) 35:30–47. doi: 10.1097/FTD.0b013e31827ada88, PMID: 23318278 PMC3546316

[56] Gunning B Mazurkiewicz-Bełdzińska M Chin RFM Bhathal H Nortvedt C Dunayevich E . Cannabidiol in conjunction with clobazam: analysis of four randomized controlled trials. Acta Neurol Scand. (2021) 143:154–63. doi: 10.1111/ane.13351, PMID: 32969022 PMC7821324

[57] Lattanzi S Trinka E Striano P Zaccara G Del Giovane C Nardone R . Cannabidiol efficacy and clobazam status: a systematic review and meta-analysis. Epilepsia. (2020) 61:1090–8. doi: 10.1111/epi.16546 32452532

[58] Szaflarski JP Hernando K Bebin EM Gaston TE Grayson LE Ampah SB . Higher cannabidiol plasma levels are associated with better seizure response following treatment with a pharmaceutical grade cannabidiol. Epilepsy Behav. (2019) 95:131–6. doi: 10.1016/j.yebeh.2019.03.042, PMID: 31048098

[59] GBD 2016 Epilepsy Collaborators. Global, regional, and national burden of epilepsy, 1990-2016: a systematic analysis for the global burden of disease study 2016. Lancet Neurol. (2019) 18:357–75. doi: 10.1016/s1474-4422(18)30454-x, PMID: 30773428 PMC6416168

