# Supplementary material for: Comparison of various doses of oral cannabidiol for treating refractory epilepsy indications: a network meta-analysis
Source: Front Neurol. 2024 Jun 27;15:1243597. doi: 10.3389/fneur.2024.1243597 (PMC11238246; doi:10.3389/fneur.2024.1243597)
Supplement: Supplementary file 1 [file Table_1.DOCX]

**Supplementary Table 1.** Detailed search strategy for each target database.

*PubMed*

| #No. | Search Details | Results |
| --- | --- | --- |
| #20 | #19 AND (humans[Filter]) | 105 |
| #19 | #15 and #18 | 120 |
| #18 | #16 or #17 | 1,699,771 |
| #17 | "random*"[All Fields] OR "placeboes"[All Fields] OR "placebos"[MeSH Terms] OR "placebos"[All Fields] OR "placebo"[All Fields] | 1,698,286 |
| #16 | "Randomized Controlled Trial"[Publication Type] OR "Randomized Controlled Trials as Topic"[MeSH Terms] OR "Random Allocation"[MeSH Terms] OR "Placebos"[MeSH Terms] | 848,960 |
| #15 | #13 or #15 | 39,076 |
| #14 | "Cannabidiol"[Title/Abstract] OR "Cannabis"[Title/Abstract] OR "medical cannabis"[Title/Abstract] OR "CBD"[Title/Abstract] | 33,224 |
| #13 | "Cannabidiol"[MeSH Terms] OR "Cannabis"[MeSH Terms] OR "Medical Marijuana"[MeSH Terms] | 16,440 |
| #12 | #3 or #8 or #11 | 40,871 |
| #11 | #9 or #10 | 17,291 |
| #10 | "lennox gastaut syndrome"[Title/Abstract] OR "dravet syndrome"[Title/Abstract] OR "tuberous sclerosis"[Title/Abstract] | 11,582 |
| #9 | "Lennox Gastaut Syndrome"[MeSH Terms] OR "epilepsies, myoclonic"[MeSH Terms] OR "Tuberous Sclerosis"[MeSH Terms] | 12,341 |
| #8 | #6 and #8 | 24,667 |
| #7 | "drug resistant"[Title/Abstract] OR "medication resistant"[Title/Abstract] OR "Intractable"[Title/Abstract] OR "drug refractory"[Title/Abstract] OR "Refractory"[Title/Abstract] | 231,155 |
| #6 | #4 or #5 | 253,320 |
| #5 | "seizure"[Title/Abstract] OR "seizures"[Title/Abstract] OR "Epilepsy"[Title/Abstract] OR "Epilepsies"[Title/Abstract] | 207,621 |
| #4 | "Epilepsy"[MeSH Terms] OR "Seizures"[MeSH Terms] | 177,848 |
| #3 | #1 or #2 | 13,581 |
| #2 | "drug resistant epilepsy"[Title/Abstract] OR "drug resistant epilepsies"[Title/Abstract] OR "medication resistant epilepsy"[Title/Abstract] OR "medication resistant epilepsies"[Title/Abstract] OR "intractable epilepsy"[Title/Abstract] OR "intractable epilepsies"[Title/Abstract] OR "refractory epilepsy"[Title/Abstract] OR "refractory epilepsies"[Title/Abstract] OR "drug refractory epilepsy"[Title/Abstract] OR "drug refractory epilepsies"[Title/Abstract] | 12,226 |
| #1 | "Drug Resistant Epilepsy"[MeSH Terms] | 3,579 |

*EMBASE*

| #No. | Search Details | Results |
| --- | --- | --- |
| #28 | #27 AND [embase]/lim AND 'human'/de | 269 |
| #27 | #15 AND #20 AND #26 | 283 |
| #26 | #21 OR #22 OR #23 OR #24 OR #25 | 2,345,049 |
| #25 | 'placebo'/exp | 398,100 |
| #24 | 'randomization'/exp | 96,147 |
| #23 | 'randomized controlled trial (topic)'/exp | 242,479 |
| #22 | 'randomized controlled trial'/exp | 747,627 |
| #21 | random*:ti,ab,kw OR placebo:ti,ab,kw | 1,998,581 |
| #20 | #16 OR #17 OR #18 OR #19 | 73,853 |
| #19 | 'medical cannabis'/exp | 3,724 |
| #18 | 'cannabis'/exp | 44,024 |
| #17 | 'cannabidiol'/exp | 7,964 |
| #16 | cannabidiol:ti,ab,kw OR cannabis:ti,ab,kw OR 'medical cannabis':ti,ab,kw OR cbd:ti,ab,kw | 48,754 |
| #15 | #3 OR #9 OR #14 | 67,273 |
| #14 | #10 OR #11 OR #12 OR #13 | 27,246 |
| #13 | 'myoclonus epilepsy'/exp | 7,468 |
| #12 | 'lennox gastaut syndrome'/exp | 4,448 |
| #11 | 'tuberous sclerosis'/exp | 12,891 |
| #10 | 'lennox gastaut syndrome':ti,ab,kw OR 'myoclonus epilepsy':ti,ab,kw OR 'tuberous sclerosis':ti,ab,kw | 16,078 |
| #9 | #7 AND #8 | 42,096 |
| #8 | 'drug resistant':ti,ab,kw OR 'medication resistant':ti,ab,kw OR intractable:ti,ab,kw OR 'drug refractory':ti,ab,kw OR refractory:ti,ab,kw | 361,525 |
| #7 | #4 OR #5 OR #6 | 441,052 |
| #6 | 'seizure'/exp | 180,427 |
| #5 | 'epilepsy'/exp | 286,652 |
| #4 | seizure:ti,ab,kw OR seizures:ti,ab,kw OR epilepsy:ti,ab,kw OR epilepsies:ti,ab,kw | 307,765 |
| #3 | #1 OR #2 | 21,779 |
| #2 | 'drug resistant epilepsy'/exp | 7,501 |
| #1 | 'drug resistant epilepsy':ti,ab,kw OR 'drug resistant epilepsies':ti,ab,kw OR 'medication resistant epilepsy':ti,ab,kw OR 'medication resistant epilepsies':ti,ab,kw OR 'intractable epilepsy':ti,ab,kw OR 'intractable epilepsies':ti,ab,kw OR 'refractory epilepsy':ti,ab,kw OR 'refractory epilepsies':ti,ab,kw OR 'drug refractory epilepsy':ti,ab,kw OR 'drug refractory epilepsies':ti,ab,kw | 19,767 |

*Cochrane library*

| #No. | Search Details | Results |
| --- | --- | --- |
| #1 | (Drug Resistant Epilepsy):ti,ab,kw OR (Drug Resistant Epilepsies):ti,ab,kw OR (Medication Resistant Epilepsy):ti,ab,kw OR (Medication Resistant Epilepsies):ti,ab,kw OR (Intractable Epilepsy):ti,ab,kw | 1,044 |
| #2 | (Intractable Epilepsies):ti,ab,kw OR (Refractory Epilepsy):ti,ab,kw OR (Refractory Epilepsies):ti,ab,kw OR (Drug Refractory Epilepsy):ti,ab,kw OR (Drug Refractory Epilepsies):ti,ab,kw | 1,123 |
| #3 | #1 or #2 | 1,868 |
| #4 | MeSH descriptor: [Drug Resistant Epilepsy] explode all trees | 156 |
| #5 | #3 or #4 | 1,868 |
| #6 | (seizure):ti,ab,kw OR (seizures):ti,ab,kw OR (Epilepsy):ti,ab,kw OR (Epilepsies):ti,ab,kw | 13,382 |
| #7 | MeSH descriptor: [Epilepsy] explode all trees | 2,635 |
| #8 | MeSH descriptor: [Seizures] explode all trees | 1,267 |
| #9 | #6 or #7 or #8 | 13,461 |
| #10 | (Resistant):ti,ab,kw OR (Intractable):ti,ab,kw OR (Refractory):ti,ab,kw | 46,770 |
| #11 | #9 and #10 | 2,449 |
| #12 | (Lennox Gastaut Syndrome):ti,ab,kw OR (Myoclonic Epilepsies):ti,ab,kw OR (Tuberous Sclerosis):ti,ab,kw | 664 |
| #13 | MeSH descriptor: [Lennox Gastaut Syndrome] explode all trees | 41 |
| #14 | MeSH descriptor: [Epilepsies, Myoclonic] explode all trees | 84 |
| #15 | MeSH descriptor: [Tuberous Sclerosis] explode all trees | 75 |
| #16 | #12 or #13 or #14 or #15 | 678 |
| #17 | #5 or #11 or #16 | 2,932 |
| #18 | (Cannabidiol):ti,ab,kw OR (Cannabis):ti,ab,kw OR (Medical cannabis):ti,ab,kw OR (CBD):ti,ab,kw | 4,159 |
| #19 | MeSH descriptor: [Cannabidiol] explode all trees | 242 |
| #20 | MeSH descriptor: [Cannabis] explode all trees | 406 |
| #21 | MeSH descriptor: [Medical Marijuana] explode all trees | 26 |
| #22 | #18 or #19 or #20 or #21 | 4,160 |
| #23 | #17 and #22 in Trials | 130 |

*Web of Science*

| #No. | Search Details | Results |
| --- | --- | --- |
|  |  |  |
| #1 | Seizure (Topic) OR seizures (Topic) OR Epilepsy (Topic) OR Epilepsies (Topic) | 385,288 |
| #2 | Resistant (Topic) OR Intractable (Topic) OR Refractory (Topic) | 1,711,023 |
| #3 | #2 AND #1 | 41,590 |
| #4 | ((TS=(Myoclonic Epilepsies )) OR TS=(Myoclonic)) OR TS=(Tuberous Sclerosis) | 31,421 |
| #5 | #4 OR #3 | 70,152 |
| #6 | (((TS=(Cannabidiol)) OR TS=(Cannabis)) OR TS=(Medical cannabis)) OR TS=(CBD) | 67,760 |
| #7 | (TS=(random*)) OR TS=(placebo) | 3,163,423 |
| #8 | #5 AND #6 AND #7 | 148 |
