# Supplementary material for: Comparison of various doses of oral cannabidiol for treating refractory epilepsy indications: a network meta-analysis
Source: Front Neurol. 2024 Jun 27;15:1243597. doi: 10.3389/fneur.2024.1243597 (PMC11238246; doi:10.3389/fneur.2024.1243597)
Supplement: Supplementary file 2 [file Table_2.DOCX]

**Supplementary Table 2.** Transitivity assessment based on 5 major factors.

| Variable | Comparison | Mean | SD | p-value |
| --- | --- | --- | --- | --- |
| Percentage of males, % | Placebo *vs.* CBD5 | 5.500 | 9.964 | 0.605 |
|  | Placebo *vs.* CBD10 | 10.833 | 6.493 | 0.139 |
|  | Placebo *vs.* CBD20 | 4.500 | 5.917 | 0.466 |
|  | Placebo *vs.* CBD25 | -1.500 | 9.964 | 0.896 |
|  | Placebo *vs.* CBD50 | -3.500 | 9.964 | 0.740 |
|  | CBD5 *vs.* CBD10 | 5.333 | 10.477 | 0.661 |
|  | CBD5 *vs.* CBD20 | -1.000 | 11.411 | 0.934 |
|  | CBD5 *vs.* CBD25 | -7.000 | n.a. | n.a. |
|  | CBD5 *vs.* CBD50 | -9.000 | n.a. | n.a. |
|  | CBD10 *vs.* CBD20 | -6.333 | 7.295 | 0.419 |
|  | CBD10 *vs.* CBD25 | -12.333 | 10.477 | 0.360 |
|  | CBD10 *vs.* CBD50 | -14.333 | 10.477 | 0.305 |
|  | CBD20 *vs.* CBD25 | -6.000 | 11.411 | 0.627 |
|  | CBD20 *vs.* CBD50 | -8.000 | 11.411 | 0.522 |
|  | CBD25 *vs.* CBD50 | -2.000 | n.a. | n.a. |
| Mean age, years | Placebo *vs.* CBD5 | 4.117 | 3.608 | 0.306 |
|  | Placebo *vs.* CBD10 | 0.650 | 2.550 | 0.806 |
|  | Placebo *vs.* CBD20 | -0.523 | 2.092 | 0.808 |
|  | Placebo *vs.* CBD25 | -0.283 | 3.608 | 0.940 |
|  | Placebo *vs.* CBD50 | 1.117 | 3.608 | 0.769 |
|  | CBD5 *vs.* CBD10 | -3.467 | 4.846 | 0.549 |
|  | CBD5 *vs.* CBD20 | -4.640 | 3.934 | 0.304 |
|  | CBD5 *vs.* CBD25 | -4.400 | n.a. | n.a. |
|  | CBD5 *vs.* CBD50 | -3.000 | n.a. | n.a. |
|  | CBD10 *vs.* CBD20 | -1.173 | 2.778 | 0.687 |
|  | CBD10 *vs.* CBD25 | 0.933 | 4.846 | 0.865 |
|  | CBD10 *vs.* CBD50 | 0.467 | 4.846 | 0.932 |
|  | CBD20 *vs.* CBD25 | 0.240 | 3.934 | 0.954 |
|  | CBD20 *vs.* CBD50 | 1.640 | 3.934 | 0.698 |
|  | CBD25 *vs.* CBD50 | 1.400 | n.a. | n.a. |
| No. of concomitant AEDs, n | Placebo *vs.* CBD5 | 0.233 | 0.390 | 0.576 |
|  | Placebo *vs.* CBD10 | -0.100 | 0.220 | 0.664 |
|  | Placebo *vs.* CBD20 | -0.127 | 0.167 | 0.468 |
|  | Placebo *vs.* CBD25 | 0.400 | n.a. | n.a. |
|  | Placebo *vs.* CBD50 | 0.400 | n.a. | n.a. |
|  | CBD5 *vs.* CBD10 | -0.333 | 0.133 | 0.130 |
|  | CBD5 *vs.* CBD20 | -0.362 | 0.052 | 0.230 |
|  | CBD5 *vs.* CBD25 | 0.400 | n.a. | n.a. |
|  | CBD5 *vs.* CBD50 | 0.400 | n.a. | n.a. |
|  | CBD10 *vs.* CBD20 | -0.027 | 0.072 | 0.725 |
|  | CBD10 *vs.* CBD25 | -0.067 | 0.133 | 0.667 |
|  | CBD10 *vs.* CBD50 | -0.067 | 0.133 | 0.667 |
|  | CBD20 *vs.* CBD25 | -0.038 | 0.048 | 0.442 |
|  | CBD20 *vs.* CBD50 | -0.038 | 0.048 | 0.442 |
|  | CBD25 *vs.* CBD50 | 0.000 | n.a. | n.a. |
| Percentage of Clobazam, % | Placebo *vs.* CBD5 | -4.817 | 15.046 | 0.762 |
|  | Placebo *vs.* CBD10 | -9.450 | 9.579 | 0.357 |
|  | Placebo *vs.* CBD20 | -2.237 | 7.342 | 0.768 |
|  | Placebo *vs.* CBD25 | 32.483 | 15.046 | 0.083 |
|  | Placebo *vs.* CBD50 | 29.183 | 15.046 | 0.110 |
|  | CBD5 *vs.* CBD10 | -4.633 | 14.476 | 0.779 |
|  | CBD5 *vs.* CBD20 | 2.580 | 10.292 | 0.814 |
|  | CBD5 *vs.* CBD25 | 37.300 | n.a. | n.a. |
|  | CBD5 *vs.* CBD50 | 34.000 | n.a. | n.a. |
|  | CBD10 *vs.* CBD20 | 7.213 | 7.702 | 0.385 |
|  | CBD10 *vs.* CBD25 | 41.933 | 14.476 | 0.101 |
|  | CBD10 *vs.* CBD50 | 38.633 | 14.476 | 0.116 |
|  | CBD20 *vs.* CBD25 | 34.720 | 10.292 | **0.028** |
|  | CBD20 *vs.* CBD50 | 31.420 | 10.292 | **0.038** |
|  | CBD25 *vs.* CBD50 | -0.340 | n.a. | n.a. |
| Treatment duration, weeks | Placebo *vs.* CBD5 | 1.000 | 1.807 | 0.604 |
|  | Placebo *vs.* CBD10 | 1.000 | 1.000 | 0.351 |
|  | Placebo *vs.* CBD20 | 0.200 | 1.045 | 0.852 |
|  | Placebo *vs.* CBD25 | -1.000 | 1.807 | 0.604 |
|  | Placebo *vs.* CBD50 | -1.000 | 1.807 | 0.604 |
|  | CBD5 *vs.* CBD10 | 0.000 | n.a. | n.a. |
|  | CBD5 *vs.* CBD20 | -0.800 | 1.960 | 0.704 |
|  | CBD5 *vs.* CBD25 | -2.000 | n.a. | n.a. |
|  | CBD5 *vs.* CBD50 | -2.000 | n.a. | n.a. |
|  | CBD10 *vs.* CBD20 | -0.800 | 1.067 | 0.482 |
|  | CBD10 *vs.* CBD25 | -2.000 | n.a. | n.a. |
|  | CBD10 *vs.* CBD50 | -2.000 | n.a. | n.a. |
|  | CBD20 *vs.* CBD25 | -1.200 | 1.960 | 0.573 |
|  | CBD20 *vs.* CBD50 | 0.000 | n.a. | n.a. |
|  | CBD25 *vs.* CBD50 | 0.000 | n.a. | n.a. |

CBD, cannabidiol; AEDs, antiepileptic drugs; SD, standard deviation; n.a., not applicable.
