# Supplementary figures and images for: Comparison of various doses of oral cannabidiol for treating refractory epilepsy indications: a network meta-analysis
Source: Front Neurol. 2024 Jun 27;15:1243597. doi: 10.3389/fneur.2024.1243597 (PMC11238246; doi:10.3389/fneur.2024.1243597)

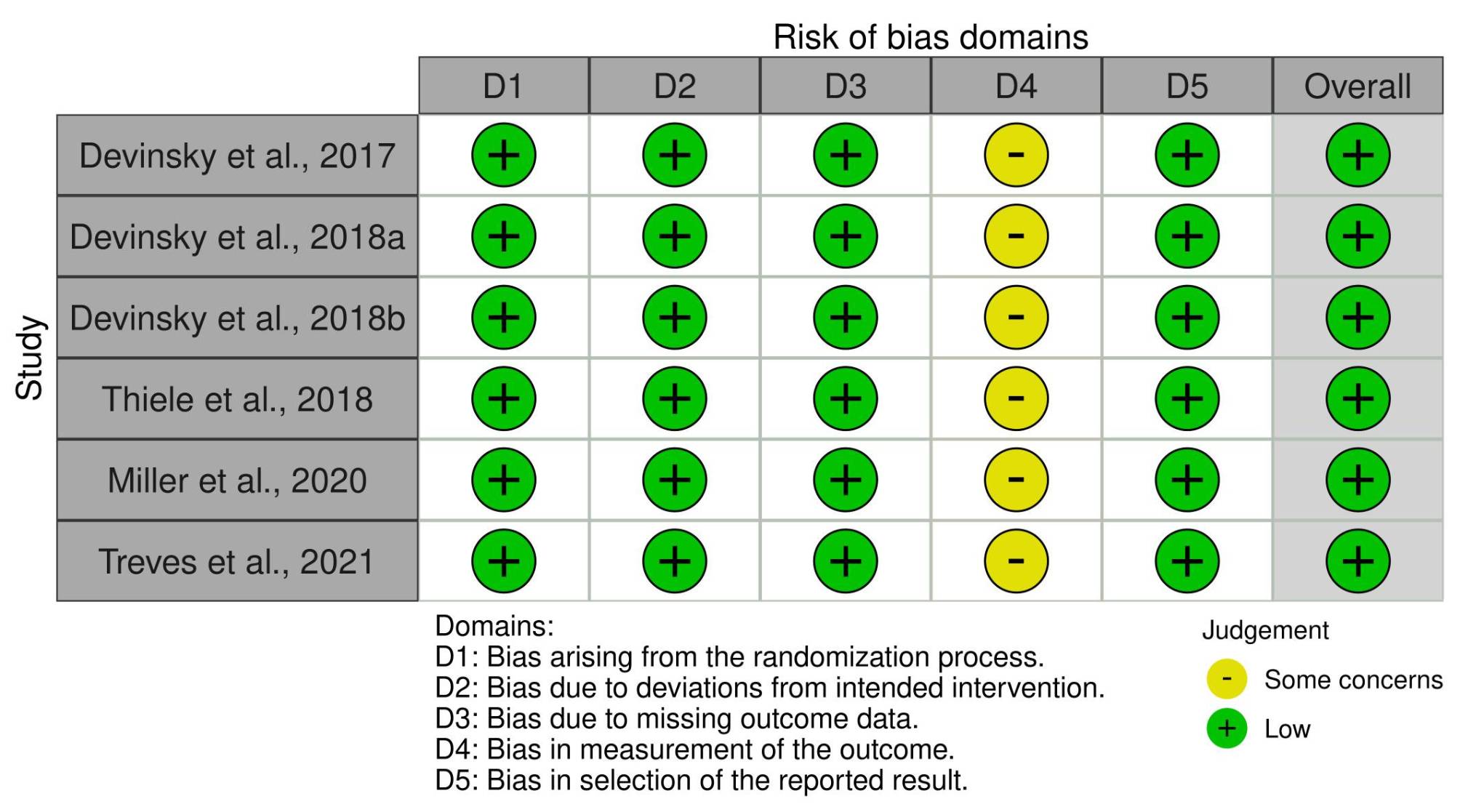

Supplement: SUPPLEMENTARY FIGURE S1 — Detailed results of the risk of bias assessment for all eligible studies. [file Image_1.JPEG]

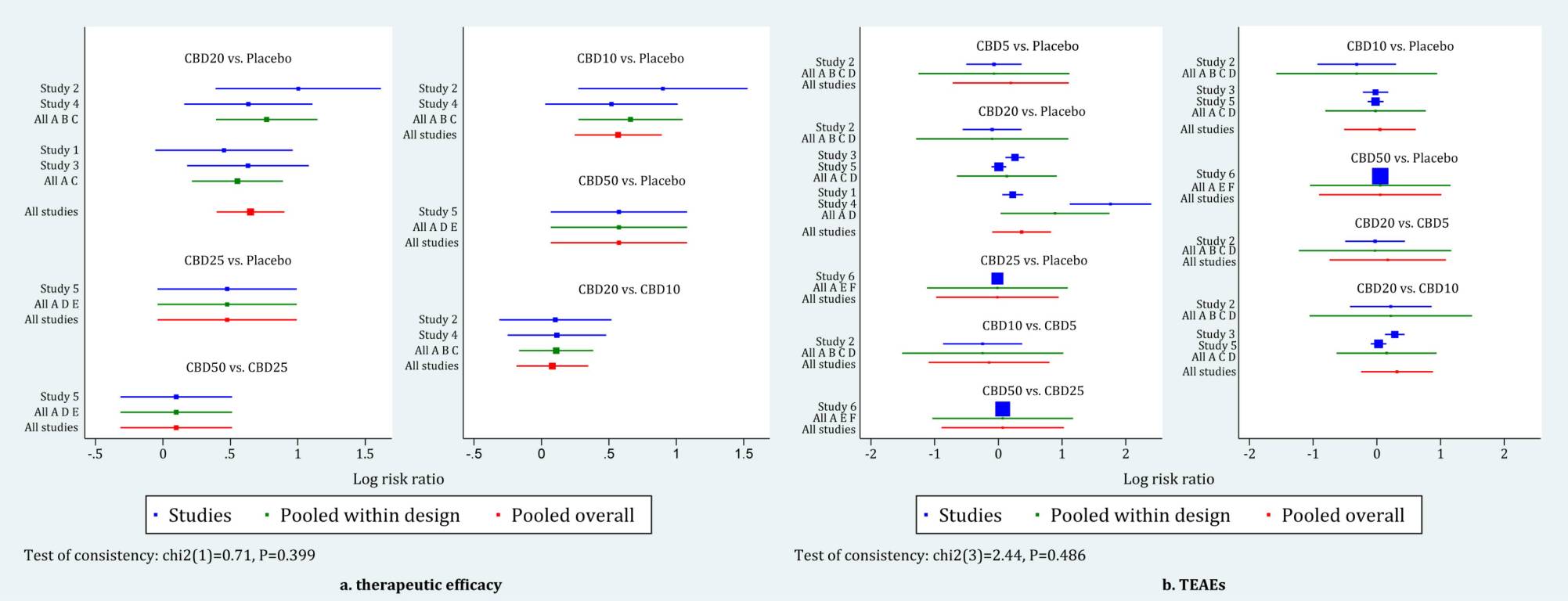

Supplement: SUPPLEMENTARY FIGURE S2 — Global inconsistency test for therapeutic efficacy (a) and TEAEs (b). CBD, cannabidiol; TEAEs, treatment-emergent adverse events. [file Image_2.JPEG]

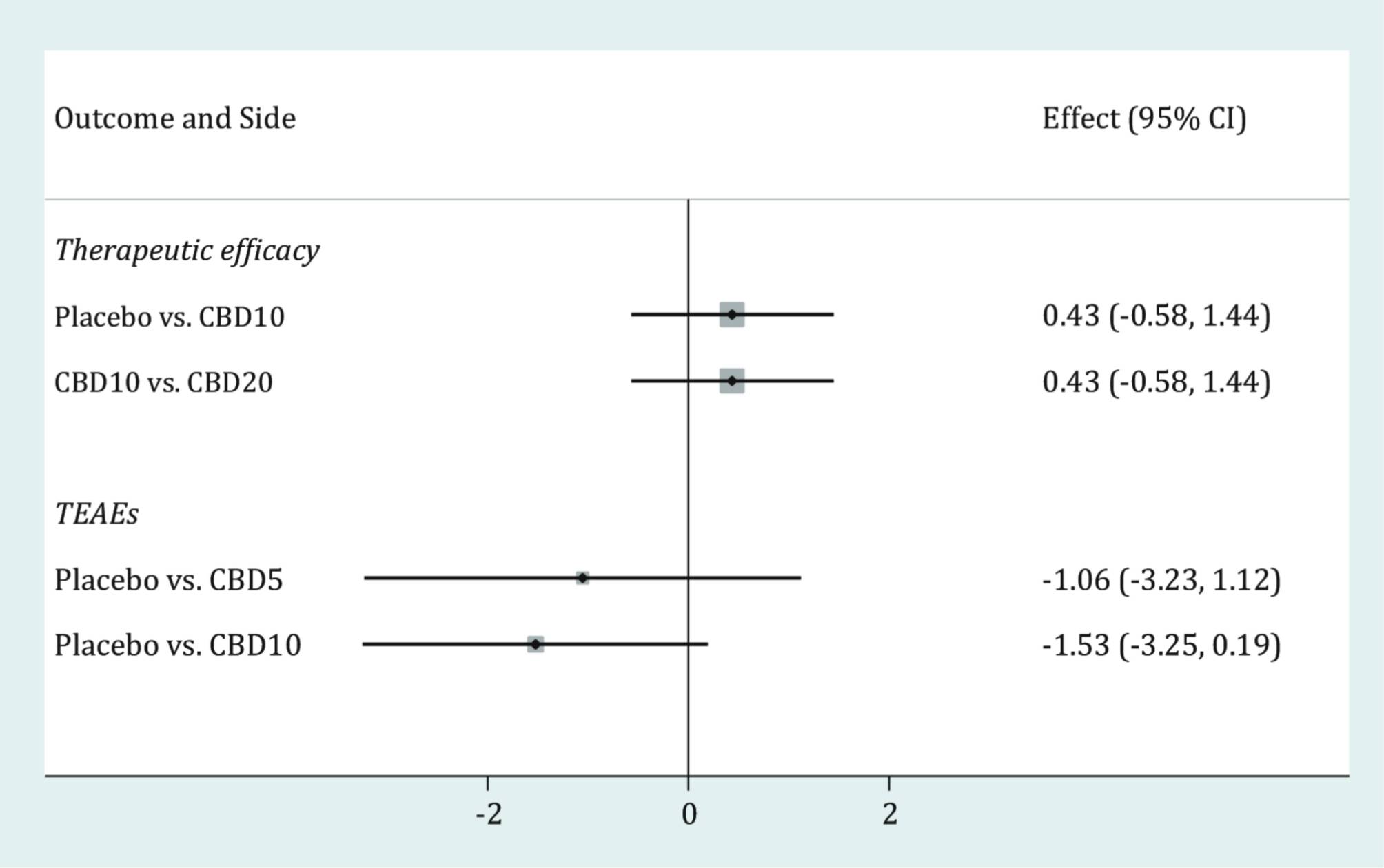

Supplement: SUPPLEMENTARY FIGURE S3 — Local inconsistency test for therapeutic efficacy and TEAEs. CBD, cannabidiol; TEAEs, treatment-emergent adverse events; CI, confidence interval. [file Image_3.JPEG]

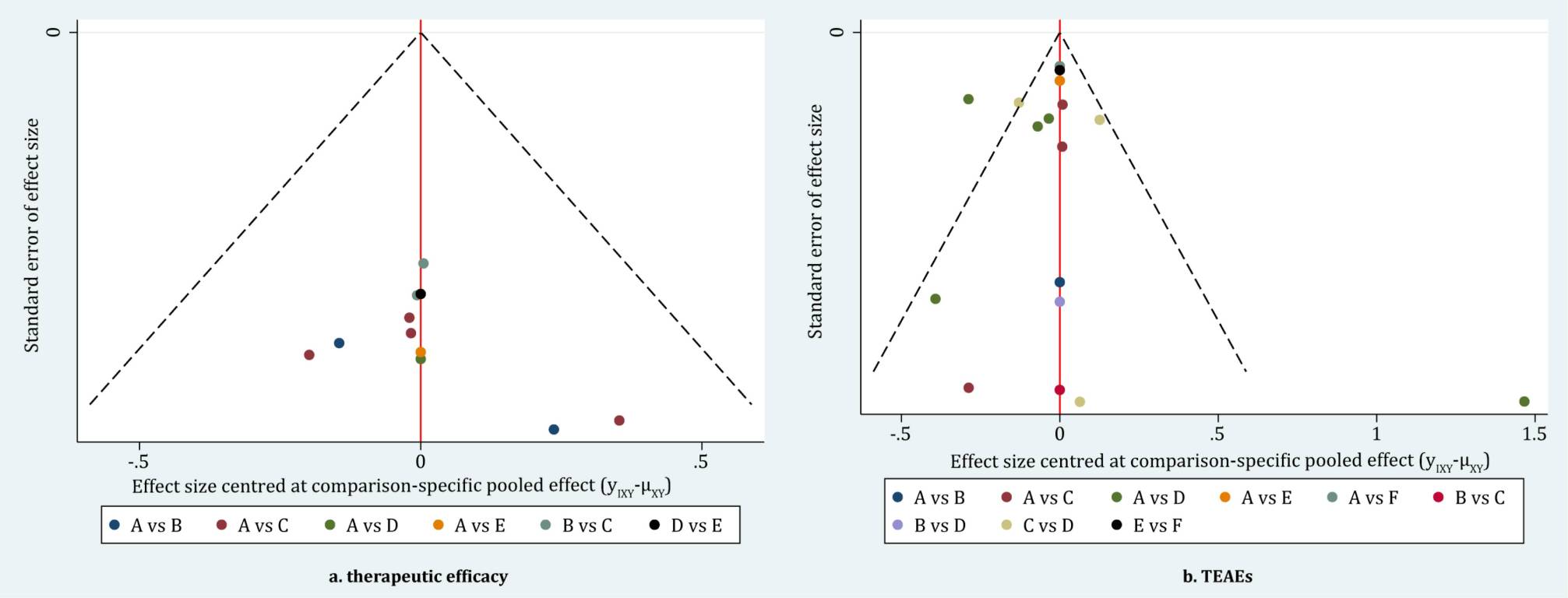

Supplement: SUPPLEMENTARY FIGURE S4 — Comparison-adjusted funnel plot for therapeutic efficacy (a) and TEAEs (b). TEAEs, treatment-emergent adverse events. [file Image_4.JPEG]
